# Assessing Health Equity in Partnership with Children’s Mental Health Organizations: Considerations Before the Implementation of Parenting Programs

**DOI:** 10.1089/heq.2023.0143

**Published:** 2024-06-27

**Authors:** Meghan Kenny, Rob Raos, Fatima Ahmad, Andrea Gonzalez

**Affiliations:** ^1^ Department of Health Research Methods, Evidence & Impact, McMaster University, Hamilton, Canada.; ^2^ Geographic Information Systems Analyst, McMaster University, Hamilton, Canada.; ^3^ Department of Psychiatry and Behavioural Neurosciences, McMaster University, Hamilton, Canada.; ^4^ Offord Centre for Child Studies, Hamilton, Canada.

**Keywords:** health impact assessment, health equity, parenting programs, children’s mental health

## Abstract

**Objectives:**

Understanding and addressing how an individual’s social, political, economic, and cultural context affects their ability to achieve optimal health is essential to designing and implementing interventions. Before evaluating two parenting programs, in partnership with four children’s mental health organizations, we used the Health Equity Impact Assessment tool (HEIA) to identify groups that may experience unintended health impacts, as well as generated mitigation strategies to address these impacts.

**Methods:**

HEIA activities included a review of the published literature, a review of organizational documents, key informant interviews with staff (*n* = 12) and other related community service providers (*n* = 7), and a geographic information systems analysis. All sources of evidence were considered and analyzed using reflective thematic analysis. Summary reports were shared with all partners.

**Results:**

A range of groups were identified as at risk of experiencing unintended health impacts, including caregivers who are racialized, immigrants, Indigenous, living with mental health issues or addictions, dealing with intellectual challenges and/or low literacy levels, survivors of childhood trauma, single parent families, or families experiencing financial difficulties. Unintended health impacts were sorted into 6 main themes which fell under the overarching themes of accessibility of the programs and cultural appropriateness. Mitigation strategies as well as innovative strategies already being applied by participating organizations are discussed.

**Conclusion:**

Although this HEIA focused on parenting programs, the findings address equity issues applicable to the provision of a wide spectrum of children’s mental health services.

## Introduction

In 2012, the government of Canada along with other World Health Organization (WHO) member states, endorsed the Rio Political Declaration on the Social Determinants of Health (SDoH)^1^ by pledging to promote health equity and strengthen capacity to monitor, assess and report on health inequities in public health policies and programs. The SDoH are factors that affect the health of individuals and communities and can lead to differential health inequities and consequences.^2^ The major causes of health inequities arise from the conditions in which people are born, grow, live, work, and age. These conditions can be social, economic, political, and/or cultural in nature. For example, individuals experiencing disadvantaged socioeconomic conditions (e.g., inadequate housing, limited access to social supports) can have different health outcomes (e.g., higher mortality rate) compared with individuals not in those conditions.

Health equity can be defined as a principle where all individuals receive support proportionate to their needs to ensure fair distribution of resources, access to opportunities, and supports offered.^3^ Health equity is achieved when there is no difference in an individual’s opportunity to obtain good health. The Ontario Ministry of Health and Long-Term Care (MOHLTC) developed a Health Equity Impact Assessment (HEIA) to support health equity, including the reduction of avoidable health disparities between population groups.^4^ The HEIA is a tool that assesses how programs and policies support health equity by identifying and addressing any unintended health impacts that a program or policy might have on marginalized groups. Specifically, the four key objectives of the HEIA^4^ are to (1) help identify unintended potential health equity impacts of decision making (positive and negative) on specific population groups; (2) support equity-based improvements in policy, planning, program, or service design; (3) embed equity in an organization’s decision-making processes; and (4) build capacity and raise awareness about health equity throughout the organization.

In Manitoba, Canada, an equity focused health impact assessment was used with success in the evaluation of an evidence-based program for caregiver of teenagers, called Teen Triple P. Several potential inequitable impacts were identified, and five key recommendations were made to address issues related to (1) the cultural appropriateness of the program, (2) agency resources, (3) supporting literacy challenges, (4) dealing with families’ broader referral and access needs, and (5) sufficient funding to address barriers such as transportation and food access.^5^

In this study, HEIAs were conducted as part of the formative evaluation for the Promoting Health Families Study (PHF). The PHF is a three-armed randomized controlled trial, for caregivers of 2–6-year-old children, examining the implementation and effectiveness of two group-based parenting programs, the Positive Parenting Program (Triple P), Group Level 4, and the Circle of Security Parenting program (COSP).^6–10^ Despite the promising findings of both programs, there is a paucity of research examining whether delivery of these programs may compromise health equity during implementation. The purpose of this study was to conduct individual HEIAs with each implementation organization with the intention of identifying any groups that may experience unintended health impacts as a result of offering these interventions in the participating communities, including families with diverse backgrounds (e.g., racialized or socially disadvantaged populations), determining what those potential unintended impacts may be (positive or negative), and generating strategies based on the evidence to mitigate these impacts.

## Methods

All participants provided written informed consent for participation, and the approval from the McMaster Ethics Board (#10583) granted ethical approval. The HEIA, developed by the MOHLTC, was the framework used to guide this study.^4^ As outlined in more detail below, the framework consisted of a review of the literature, key informant interviews, agency documentation reviews, and a geographic information systems analysis.

## Data Sources

### Participants, Setting, and Key Informant Interviews

HEIAs were conducted with four children’s mental health agencies in southern Ontario, covering a broad geographic area. Key informant interviews were conducted via Zoom with staff members (*n* = 12) from each agency. These staff members ranged from executive directors, directors of clinical services, managers of intervention services, and clinicians facilitating groups. Interviews were informed by the guides provided in the Ministry’s HEIA handbook^4^ to understand each agency’s efforts to address health equity in their provision of mental health services.

Additional interviews (*n* = 5) were conducted with partner organizations identified by the participating agencies. These partner organizations were familiar with the services provided by the agencies, as well as the unique needs of their communities. These organizations included point of access agencies for child-focused services/supports, child protection agencies, and community health centers. Community practitioners (*n* = 2) with considerable experience working with Indigenous families and delivering group-based parenting programs were also consulted. Finally, representatives from both parenting programs (*n* = 2) were interviewed to understand their perspective on the strengths and shortcomings of their respective programs in relation to health equity.

### Literature Review

We conducted a review of English publications focused on health equity in relation to children’s mental health services and, more specifically, on parenting programs for caregivers of young children. These findings were used to determine the SDoHs and health inequities experienced by various marginalized groups identified through key informant interviews. Included in the search were peer-reviewed articles, systematic reviews, and gray literature, such as government reports and internal documents from the participating agencies.

### Agency Document Review

We requested internal documents from each of the participating agencies. A total of 39 documents were reviewed by the research team. These included clinical policy and procedure manuals, annual reports, strategic plans, program handbooks, past presentations, and memorandums of understanding with other community organizations. Information appraised for this evaluation was related to policies, protocols, and procedures pertaining to health equity and providing support services to families from marginalized groups.

### Geographic Information Systems (GIS) Analysis

A GIS Research Analyst conducted a detailed description of the each of the catchment areas, defined using Forward Sortation Areas, to gain a better sense of the ethnic composition and socioeconomic status of each of the participating agencies’ catchment areas. Data from the 2016 Census at the Dissemination Area (DA) level were linked to identified catchment areas. Census variables of interested were retrieved at the DA level and aggregated to provide measures for each catchment area. In addition to the Census, measures from the Ontario Marginalization Index were compiled for the catchment areas by aggregating data at the DA level. Two dimensions relevant to the HEIA were extracted from the Ontario Marginalization Index: material deprivation and ethnic concentration.^11^ Material deprivation is closely connected to poverty, and it refers to inability for individuals and communities to access and attain basic material needs. The indicators included in this dimension measure income, quality of housing, educational attainment, and family structure characteristics. Ethnic concentration refers to high area-level concentrations of people who are recent immigrants and/or people belonging to a “visible minority” group (defined by Statistics Canada as “persons, other than aboriginal peoples, who are non-Caucasian in race or non-white in colour”). Both the material deprivation and ethnic concentration dimensions are scored based on the quintile distribution of the measure across Ontario, where 1 is the least deprived/ethnically concentrated and 5 is the most deprived/ethnically concentrated.

### HEIA Analysis

All sources of evidence were considered in the HEIA analyses with the various themes summarized in the categories provided in the HEIA template (see Supplementary Appendix A1). The main categories included are:

1. Scoping – populations and determinants of health are identified.
2. Potential Impacts – unintended impacts (positive and negative) are reported.
3. Mitigation – recommendations for addressing potential health impacts are outlined.
4. Monitoring – where steps for measuring impact of mitigation strategies are detailed.
5. Dissemination – ways of sharing findings and recommendations are identified.

Individual reports were presented to each agency, and discussions were held with representatives to review the recommendations, as well as the feasibility of implementing proposed mitigation strategies.

## Findings

### Geographic Information Systems Data Summary

The combined populations of the cities of the four agency catchments were approximately 3,263,000. Two agencies serve a predominantly urban catchment, with 98.79% of DAs in one catchment reporting a population density above 400 people per square kilometer and the other reporting 99.67%. Two agencies serve catchments composed of both urban and rural settings, with 77.05% of the DAs in one catchment reporting a population density above 400 people per square kilometer and the other reporting 83.44%. Material deprivation scores for all study sites skew toward higher deprivation than the average amounts reported for Ontario (with aggregated quintiles varying from 2.99 to 3.37). All agencies serve racially and ethnically diverse communities. The aggregated quintiles for ethnic concentration across sites varied from 2.27 to 3.99 (with higher numbers indicating greater ethnic diversity).

### Summary of Potential Unintended Health Impacts and Recommended Mitigation Strategies

There were several groups identified as potentially experiencing unintended health impacts because of offering these parenting interventions in the focus communities. These groups included caregivers who are dealing with financial difficulties, from racialized groups, new to Canada, Indigenous, living with mental health issues or addictions, dealing with intellectual challenges and/or low literacy levels, survivors of childhood trauma, single parent families, or families facing financial difficulties. Applying reflective thematic analysis methods articulated by Braun & Clarke,^12^ there were six main themes that emerged which were categorized under two overarching themes of accessibility of the programs and cultural appropriateness. Each is discussed in greater detail below.

### Accessibility

#### Technology

Before the COVID-19 pandemic, these parenting groups would have been offered in person, at the agencies or other locations within the community. This study commenced at the beginning of the pandemic, and as a result, the parenting interventions had to be offered virtually to adhere to public health restrictions. Knowing that technology may present an immediate barrier to many families, our research team and the agencies combined resources to provide participants with access to the internet and a tablet for the duration of the study.

#### Timing of group sessions

A significant barrier to caregiver participation in programming is the time of day that services are provided.^13–18^ Many of the families being served by the agencies are led by single mothers and/or by caregivers whose jobs require shiftwork. Mornings present challenges for some caregivers, who are tasked with getting to work and/or dropping their children off to school. This was further exacerbated when parents were forced to manage working from home while providing hands-on support with their children’s online schooling during the pandemic. Evenings provided challenges for other caregivers, who were doing shiftwork or working in the evenings. As such, it was recommended that agencies offer group sessions at a variety of times and to provide short “make-up” sessions if a session was missed due to scheduling difficulties.

#### Group content

Accessibility of both the program materials and concepts was identified as potential barriers for some caregivers. It was recommended that group providers offer caregivers support outside of sessions to assist with comprehension and completion of homework. To capture the additional time spent by service providers to support caregivers, our research team integrated tracking sheets of time spent on various tasks related to implementation of the program and supporting families into the data collection.

#### Awareness of services

Concerns regarding a general lack of awareness of services in the community, particularly in the case of racialized families, were highlighted. This is widely reflected in the literature as well.^17,19,20^ To address this potential barrier, our research team and the participating agencies collaborated with a marketing consultant and their internal communications departments to design a multipronged advertising campaign. This consisted of radio and social media advertisements and posters strategically placed throughout the communities (e.g., schools, health care, and community centers). Information regarding the parenting programs was also shared broadly with community organizations and service providers that are trusted and frequented by racialized families (e.g., multicultural centers, family physicians, childcare centers, libraries, etc.).

### Cultural Appropriateness

#### Cultural appropriateness and safety of programs: Indigenous caregivers

It is well-established that there is a paucity of culturally appropriate services available to Indigenous populations.^21^ Interviews and internal documents revealed that the agencies were in varying stages of reaching and engaging Indigenous caregivers. For example, one of the agencies has developed a formal memorandum of understanding with the local Indigenous center to provide mutually beneficial services, build a more inclusive system to meet the mental health needs of children and families, and foster learning opportunities through workshops, trainings, and lectures. An Indigenous elder also works closely with that agency to advise on supporting Indigenous families in the area. Participating agencies were advised to continue to reach out to local Indigenous organizations and elders to seek guidance around delivering the parenting programs to Indigenous families and to determine the cultural appropriateness of the group content and materials.

#### Cultural appropriateness and safety of programs: Racialized caregivers

One key concern frequently identified in the health equity literature is the cultural appropriateness and safety of programming for racialized families.^15,17,22^ To create a culturally safe environment for all participants, it was recommended that group facilitators participate in coaching sessions led by a COSP representative as well as attend regular peer support meetings. The research team provided funding for COSP coaching sessions for group facilitators. COSP coaching was focused on assisting facilitators with navigating conflicts between program concepts and the cultural values of families, identifying and being mindful of their own biases, and avoiding using prescriptive approaches. The research team also funded the Triple P Ontario Network website which ensures availability to all Triple P resources and materials. In addition, an implementation specialist was available to consult with any specific questions that providers may have during program sessions.

### Agency Efforts to Address Health Inequities

Before our involvement with participating agencies, most of the study partners were at various points of planning or implementing their own strategies to address health inequities, particularly approaches related to providing services to racialized families. Some of the strategies are outlined in Table 1 below:

**Table 1: Strategies Used by Children's Mental Health Agencies to Address Health Inequities:** 

Ensuring that the racial and ethnic diversity of agency staff members reflects the diversity represented in the community.	Acknowledging the strengths and knowledge each caregiver brings from their extensive histories and backgrounds.
Emphasizing that the parenting approaches being presented in sessions are not the only or right way to parent.	Working with caregivers to identify the similarities between their culture and the concepts being introduced in groups.
When possible, organizing groups to include caregivers from similar cultural backgrounds.	Raising awareness of programs and establishing trust with racialized families by cofacilitating groups with other organizations/service providers familiar to the community.

## Discussion

Health inequities have long existed within care provision systems. An increasingly diverse demographic within Canada further raises concern for equitable access to health care and calls for immediate and sustainable mitigation strategies. The findings of HEIAs from this project highlighted inequities which fell within two overarching themes of accessibility and cultural appropriateness. Collectively, these HEIA findings highlighted similar barriers to accessing and engaging with services identified in previous literature, including logistical barriers, such as conflicting appointments, employment, lack of transport and childcare provision,^13–19^ as well as a general lack of awareness of community services paired with lower levels of health literacy.^17,19,20^ Additional barriers, ranging from individual to structural levels, include stigma, mistrust, family/community perceptions, racism and discrimination, and lack of linguistic and culturally congruent service delivery.^14,16,17,19,22^ One of the most notable findings from our study was the identification of challenges that agencies and their community partners face in engaging racialized families. Failing to address social and health inequities faced by these groups results in significant gaps in service utilization rates and overall health status.^23,24^ For example, health disparities arise due to a number of factors including geographic isolation, language barriers, social, political, and economic consequences of colonialism and lack of culturally relevant education and health delivery, which deter Indigenous people from accessing care.^21,23,24^ Similarly, lack of culturally sensitive programming that addresses social and cultural determinants of health leads to limited recruitment and engagement of racialized families in health services and research.^15,17,22^

The HEIAs also provided mitigation strategies to address the identified unintended health impacts. It is important to note that several agencies were already aware of inequities in their program delivery and were at varying points of planning and implementing strategies to address them. Recommendations to reduce accessibility-related barriers included providing technological access for virtual program delivery, increasing flexibility for group session timings, offering alternate support and make-up sessions, and placing a multipronged advertising campaign for agencies in the community. Strategies to inform culturally appropriate care included engaging with Indigenous community centers to develop mutually beneficial, inclusive, and culturally sensitive health programs for Indigenous families. To determine if the HEIA recommendations addressing accessibility and cultural appropriateness of the groups were implemented and if they impacted participants’ experience, questions have been included in the group satisfaction questionnaire as part of evaluation, which will inform step four of the HEIA framework: monitoring of strategies. Monitoring methods were also incorporated into program delivery by having group facilitators track additional time spent with caregivers outside of group on weekly basis. A question was also included in caregiver interviews in the process evaluation, where participants were asked if they were offered additional support from their group facilitator.

In sum, the HEIAs revealed numerous unintended health impacts that the two parenting programs may have on families with diverse backgrounds. The two main categories of limited accessibility and cultural appropriateness within the services are largely consistent with previous literature, pointing to a dire need to address them through equitable and sustainable policy changes. Mitigation strategies emerging from internal agency discussions and this assessment included implementing culturally sensitive training for service providers, creating a diverse and representative health care workforce, and addressing distal to proximal points of accessing health care services for racialized and Indigenous communities. The last two steps of HEIA, monitoring and disseminating the short- and long-term impact of these strategies, will ensure stakeholders and relevant partners continue to assess and revise multiple domains of Canada’s health care system to be more equitable and accessible for all communities.

## Supplementary Material

Supplementary Appendix A1

## Notes

### Abbreviations Used

COSP: Circle of Security Parenting
DA: Dissemination Area
EfHIA: Equity Focused Health Impact Assessment
GIS: Geographic Information Systems
HEIA: Health Equity Impact Assessment
MOHLTC: Ministry of Health and Long-Term Care
PHF: Promoting Health Families
RCT: Randomized Controlled Trial
SDoH: Social Determinants of Health
Triple P: Positive Parenting Program
WHO: World Health Organization

## References

[1] World Health Organization. Rio political declaration on social determinants of health. World Health Organization: Rio de Janeiro: 2011. Available from: https://iris.who.int/bitstream/handle/10665/23747/B130_15-en.pdf?sequence=1.

[2] Social determinants of health. World Health Organization: Geneva, CH; 2008. Available from: https://www.who.int/health-topics/social-determinants-of-health#tab=tab_1.

[3] Health equity guideline, 2018. Ministry of Health and Long-Term Care (MOHLTC): Ontario, Canada; 2018. Available from: https://files.ontario.ca/moh-guidelines-health-equity-guideline-en-2018.pdf.

[4] Health Equity Impact Assessment (HEIA) tool and workbook. Ministry of Health and Long-Term Care (MOHLTC): Ontario, Canada; 2012. Available from: https://www.camh.ca/-/media/files/professionals/heia/health-equity-impact-assessment-workbook2012-pdf.pdf.

[5] Cohen BE, Ateah CA, Chartier MJ, et al. Report of an equity-focused health impact assessment of a proposed universal parenting program in Manitoba. Can J Public Health 2016;107(1):e112–e118; doi: 10.17269/cjph.107.5108 27348097 PMC6972271

[6] Sanders MR. Triple P–Positive Parenting Program: A population approach to promoting competent parenting. AeJAMH 2003;2(3):127–143; doi: 10.5172/jamh.2.3.127

[7] de Graaf I, Speetjens P, Smit F, et al. Effectiveness of the Triple P Positive Parenting Program on behavioral problems in children: A meta-analysis. Behav Modif 2008;32(5):714–735; doi: 10.1177/0145445508317134 18475003

[8] Sanders MR, Kirby JN, Tellegen CL, et al. The Triple P-Positive Parenting Program: A systematic review and meta-analysis of a multi-level system of parenting support. Clin Psychol Rev 2014;34(4):337–357; doi: 10.1016/j.cpr.2014.04.003 24842549

[9] Baumann AA, Powell BJ, Kohl PL, et al. Cultural adaptation and implementation of evidence-based parent-training: A systematic review and critique of guiding evidence. Child Youth Serv Rev 2015;53:113–120; doi: 10.1016/j.childyouth.2015.03.025 25960585 PMC4419735

[10] Yaholkoski A, Hurl K, Theule J. Efficacy of the circle of security intervention: A meta-analysis. J Infant Child Adolesc Psychother 2016;15(2):95–103; doi: 10.1080/15289168.2016.1163161

[11] Matheson F, Van Ingen T. Ontario Marginalization Index: User Guide. Ontario Agency for Health Protection and Promotion (Public Health Ontario): Ontario, Canada; 2016.

[12] Braun V, Clarke V. Can I use TA? Should I use TA? Should I not use TA? Comparing reflexive thematic analysis and other pattern‐based qualitative analytic approaches. Couns Psychother Res 2021;21(1):37–47; doi: 10.1002/capr.12360

[13] Hackworth NJ, Matthews J, Westrupp EM, et al. What influences parental engagement in early intervention? parent, program and community predictors of enrolment, retention and involvement. Prev Sci 2018;19(7):880–893; doi: 10.1007/s11121-018-0897-2 29629506 PMC6182377

[14] Waheed W, Hughes-Morley A, Woodham A, et al. Overcoming barriers to recruiting ethnic minorities to mental health research: A typology of recruitment strategies. BMC Psychiatry 2015;15(1):101–111; doi: 10.1186/s12888-015-0484-z 25934297 PMC4436137

[15] Brown AF, Ma GX, Miranda J, et al. Structural interventions to reduce and eliminate health disparities. Am J Public Health 2019;109(S1):S72–S78; doi: 10.2105/ajph.2018.304844 30699019 PMC6356131

[16] Planey AM, Smith SM, Moore S, et al. Barriers and facilitators to mental health help-seeking among African American youth and their families: A systematic review study. Child Youth Serv Rev 2019;101:190–200; doi: 10.1016/j.childyouth.2019.04.001

[17] Salam Z, Odenigbo O, Newbold B, et al. Systemic and individual factors that shape mental health service usage among visible minority Immigrants and refugees in Canada: A scoping review. Adm Policy Ment Health 2022;49(4):552–574; doi: 10.1007/s10488-021-01183-x 35066740

[18] Baucom KJW, Chen XS, Perry NS, et al. Recruitment and Retention of Low-SES Ethnic Minority Couples in Intervention Research at the Transition to Parenthood. Fam Process 2018;57(2):308–323; doi: 10.1111/famp.12287 28328086 PMC7087449

[19] Thomson MS, Chaze F, George U, et al. Improving immigrant populations' access to mental health Services in Canada: A review of barriers and recommendations. J Immigr Minor Health 2015;17(6):1895–1905; doi: 10.1007/s10903-015-0175-3 25742880

[20] Axford N, Lehtonen M, Kaoukji D, et al. Engaging parents in parenting programs: Lessons from research and practice. Child Youth Serv Rev 2012;34(10):2061–2071; doi: 10.1016/j.childyouth.2012.06.011

[21] Public Health Agency of Canada. Key health inequalities in Canada: A national portrait executive summary. Ottawa, ON; 2018. Available from: https://www.canada.ca/en/public-health/services/publications/science-research-data/key-health-inequalities-canada-national-portrait-executive-summary.html.

[22] Brown G, Marshall M, Bower P, et al. Barriers to recruiting ethnic minorities to mental health research: A systematic review. Int J Methods Psychiatr Res 2014;23(1):36–48; doi: 10.1002/mpr.1434 24474683 PMC6878438

[23] Davy C, Harfield S, McArthur A, et al. Access to primary health care services for Indigenous peoples: A framework synthesis. Int J Equity Health 2016;15(1):163–169; doi: 10.1186/s12939-016-0450-5 27716235 PMC5045584

[24] Nguyen NH, Subhan FB, Williams K, et al. Barriers and mitigating strategies to healthcare access in Indigenous communities of Canada: A narrative review. Healthcare 2020;8(2); doi: 10.3390/healthcare8020112 PMC7349010 32357396

