# Supplementary material for: Assessing Health Equity in Partnership with Children’s Mental Health Organizations: Considerations Before the Implementation of Parenting Programs
Source: Health Equity. 2024 Jun 27;8(1):419–25. doi: 10.1089/heq.2023.0143 (PMC11250835; doi:10.1089/heq.2023.0143)
Supplement: Supplementary Appendix A1 [file heq.2023.0143_suppl_datas1.docx]

| **HEIA Table Template** | | | | | | | | | | |  |
| --- | --- | --- | --- | --- | --- | --- | --- | --- | --- | --- | --- |
| **Step 1.**  **SCOPING** | **Step 2.**  **POTENTIAL IMPACTS** | | | | **Step 3.**  **MITIGATION** | | **Step 4.**  **MONITORING** | | **Step 5. DISSEMINATION** | |  |
| **a) Populations***  Using evidence, identify which populations may experience significant unintended health impacts (positive or negative) as a result of the planned policy, program or initiative. | **b) Determinants of Health**  Identify determinants and health inequities to be considered alongside the populations you identify. | **Unintended Positive Impacts** | **Unintended Negative Impacts** | **More Information Needed** | | **Identify ways to reduce potential negative impacts and amplify the positive impacts** | | **Identify ways to measure success for each mitigation strategy identified** | | **Identify ways to share results and recommendations to address equity** | |
|  |  |  |  |  | |  | |  | |  | |
|  |  |  |  |  | |  | |  | |  | |
|  |  |  |  |  | |  | |  | |  | |
